# Effect of Mn^2+^ on Upconversion Emission, Thermal Sensing and Optical Heater Behavior of Yb^3+^ - Er^3+^ Codoped NaGdF_4_ Nanophosphors

**DOI:** 10.3389/fchem.2019.00425

**Published:** 2019-06-06

**Authors:** Qinping Qiang, Yuhua Wang

**Affiliations:** ^1^Department of Materials Science, School of Physical Science and Technology, Lanzhou University, Lanzhou, China; ^2^Key Laboratory for Special Function Materials and Structural Design of the Ministry of Education, Lanzhou University, Lanzhou, China

**Keywords:** NaGdF_4_:Yb^3+^/Er^3+^, Mn^2+^, upconversion luminescence, temperature sensing, optical heater

## Abstract

In thiswork, we investigate the influence of Mn^2+^ on the emission color, thermal sensing and optical heater behavior of NaGdF_4_: Yb/Er nanophosphors, which the nanoparticles were synthesized by a hydrothermal method using oleic acid as both a stabilizing and a chelating agent. The morphology and crystal size of upconversion nano particles (UCNPs) can be effectively controlled through the addition of Mn^2+^ dopant contents in NaGdF_4_: Yb/Er system. Moreover, an enhancement in overall UCL spectra of Mn^2+^ doped UCNPs for NaGdF_4_ host compared to the UCNPs is observed, which results from a closed back-energy transfer between Er^3+^ and Mn^2+^ ions (^4^S_3/2_ (Er^3+^) → ^4^T_1_ (Mn^2+^) → ^4^F_9/2_ (Er^3+^)). The temperature sensitivity of NaGdF_4_:Yb^3+^/Er^3+^ doping with Mn^2+^ based on thermally coupled levels (^2^H_11/2_ and ^4^S_3/2_) of Er^3+^ is similar to that particles without Mn^2+^ in the 303–548 K range. And the maximum sensitivity is 0.0043 K^−1^ at 523 K for NaGdF_4_:Yb^3+^/Er^3+^/Mn^2+^. Interestingly, the NaGdF_4_:Yb^3+^/Er^3+^/Mn^2+^ shows preferable optical heating behavior, which is reaching a large value of 50 K. These results indicate that inducing of Mn^2+^ ions in NaGdF_4_:Yb^3+^/Er^3+^ nanophosphors has potential in colorful display, temperature sensor.

## Introduction

Owing to the virtues of non-invasion, rapid response, high spatial resolution and signal to noise ratio, rare earth ions (Ln^3+^) doped up-conversion luminescent (UCL) material as an optical thermometer have been a subject of particular interest now a day, which can be applicable in life sciences, industrial production, aerospace and military (Fischer et al., 2011; Sedlmeier et al., 2012; Chen et al., 2013; Liu et al., 2015; Yang et al., 2015; Zheng et al., 2015). Trivalent lanthanide ion has abundant ladder-like levels, which can convert two or more low energy photons to a higher energy photon (Dong et al., 2012; Niu et al., 2012; Xu et al., 2015). Erbium ion (Er^3+^) is one of the most significant activator, whose luminescence ranges from visible to ultraviolet under near infrared (NIR) excitation (Gai et al., 2013; Wang et al., 2016).

Among temperature dependent optical performance, such as peak position (Jiang et al., 2014), luminescent lifetime (Peng et al., 2010), emission intensity (Zhou et al., 2016), and bandwidth (Walsh and Di Bartolo, 2015), fluorescence intensity ratio (FIR) technique (Liu et al., 2017; Xu et al., 2017) can achieve accurate temperature measurement, which is independent of external interferences, spectral losses, as well as fluctuations in the excitation density (Wade et al., 2003; Wawrzynczyk et al., 2012; Zhou et al., 2014; Pandey et al., 2015; Wang et al., 2015). Using this ratiometric technique, the sensitivity of sensor is strongly dependent on the energy gap of thermally coupled levels (TCL), which is confined in the range of 200–2,000 cm^−1^ (Zheng et al., 2016; Du et al., 2017; Tong et al., 2017; Wang et al., 2017). Generally, the larger energy gap of TCL leads to the higher sensitivity (Du et al., 2016). Therefore, the TCL ^2^H_11/2_ and ^4^S_3/2_ of Er^3+^ have been used in FIR thermometry due to their larger energy gap (~800 cm^−1^) (Zheng et al., 2014; Chen et al., 2017), intense green emissions and minor overlap between two green emission peaks (León-Luis et al., 2013). It has been reported that the sensing sensitivity of Er^3+^ doped up-conversion nanocrystals is mainly depended on the host matrix, exciting power and nanocrystal size (Dong et al., 2014, 2015; Marciniak et al., 2016).

Notably, the host is one of the most important factors to determine UC efficiency since the phonon energy of host has a significant impact on the probability of non-radiative transitions for the incorporated Ln^3+^ dopants (Wang and Liu, 2009). Yb/Er co-doped NaLnF_4_ (Ln = Y, La, Lu, Yb, Gd) hosts are considered as the most efficient UCL systems (Zeng et al., 2014). However, it is still a challenge to achieve multi-color output and enhanced red UCL in a single fixed composition of Yb/Er co-doped system. Recently, some dopants, such as divalent manganese (Mn^2+^), have been recognized as effective elements which can decrease the short-wavelength green emission and enhance the long-wavelength red emission because of the energy transfer between Er^3+^ and Mn^2+^ ions (Tian et al., 2012; Liu et al., 2019).

Here, we construct an energy transfer bridge to achieve high sensitivity for the temperature sensing. The hexagonal phase NaGdF_4_ is selected as the matrix material due to its low phonon energy and remarkable chemical stability. Compared with NaGdF_4_:Yb^3+^/Er^3+^ phosphor, the luminescence of NaGdF_4_:Yb^3+^/Er^3+^/Mn^2+^ phosphor is illustrated under 980 nm excitation. Importantly, the Mn^2+^ doping in phosphor could be result in energy transfer between ^4^S_3/2_ and ^4^F_9/2_ (Er^3+^). Meanwhile, the temperature sensing behaviors of two phosphors are investigated in the temperature 303–523 K based on TCL. The internal heating of the developed phosphors has been computed employing temperature dependent FIR study at the same time. The influences of energy transfer induced by Mn^2+^ ions are discussed for temperature sensing and optical heating.

## Experimental

### Synthesis of β-NaGdF_4_: 20 mol% Yb^3+^/1 mol% Er^3+^/*x* mol% Mn^2+^(0 ≤ *x* ≤ 40) Nanoparticles

NaGdF_4_: 20 mol% Yb^3+^/1 mol% Er^3+^/*x* Mn^2+^ (*x* = 0, 5, 10, 20, 30, and 40 mol%) nanocrystals were synthesized by a hydrothermal method using oleic acid as both a stabilizing and a chelating agent. The typical synthesis involved the addition of 10 mL of ethanol to 2 mL of an aqueous solution containing 1.2 g of NaOH under stirring to form a homogeneous solution. Then, 20 mL of oleic acid was added to form a sodium-oleic acid complex. Subsequently, 1 mmol RE(NO_3_)_3_ (RE = Gd, Yb, and Er with designed molar ratios) and 8 mL of 1.0 M NaF aqueous and stoichiometric ratio of Mn(NO_3_)_2_ solutions were added under constant vigorous stirring for 10–20 min. The resulting solution was transferred into a 50 mL stainless Teflon-lined autoclave, which was operated at 170°C for 24 h. After reaction completion, the system was naturally cooled to room temperature. The resulting samples were washed several times with ethanol and de-ionized water to remove oleic acid and other residual solvents, and then dried at 60°C for 10 h. The NaGdF_4_: 20 mol% Yb^3+^/1 mol% Er^3+^ nanoparticle is labeled as NaGdF_4_: Yb/Er, and the NaGdF_4_: 20 mol% Yb^3+^/1 mol% Er^3+^/*x* mol% Mn^2+^ sample is labeled as NaGdF_4_: Yb/Er/*x* Mn.

### Characterization

The X-ray diffraction (XRD) patterns were obtained on a Rigaku D/Max-2400 X-ray diffractometer with Ni-filter Cu K α radiation at 40 kV and 60 mA. The size, shape and structure of the as-prepared microcrystals were characterized by scanning electron microscopy (SEM) (S-4800), transmission electron microscopy (TEM) (JSM-1200EX) and high-resolution transmission electron microscopy (HRTEM) (FEI Tecnai F30, operated at 300 kV). The elemental analysis identified by energy dispersive X-ray spectroscopy (EDX) was attached with the same TEM. In the measurements of UC emission, a continuous 980 nm laser diode (LD) with a power maximum of 1.5 W was used for excitation sources. The samples used in the upconversion measurement and Pump Power dependence measurements are powder samples. The powder samples are pressed on a sample tray which is cover with a quartz glass sheet, then the output laser beam collimated and focused on the samples to test. All measurements were performed at room temperature.

## Results and Discussion

### Phase Identification and Crystal Structure of β-NaGdF_4_

**Figure 1:**
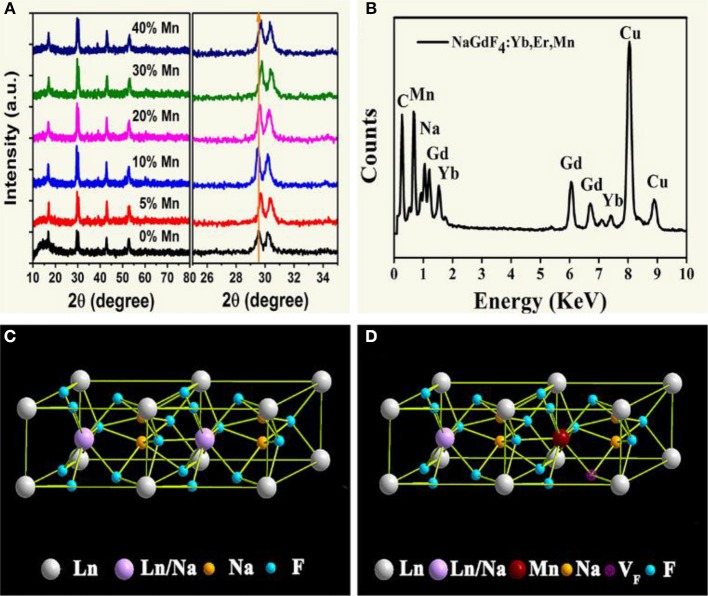
**(A)** XRD patterns of NaGdF_4_: Yb/Er/*x* Mn NCs (0 ≤ *x* ≤ 40); **(B)** EDX spectrum of the corresponding sample, all the signals are normalized to Gd one, and Cu signals come from copper grid; **(C)**, **(D)** schematic illustrations of the crystal structures for pure and Mn^2+^ doped NaGdF_4_, respectively.

The structure of all samples is typical hexagonal phase. As shown in Figure 1A, XRD studies show peak positions and intensities that can be well-indexed in accordance with β-NaGdF_4_ crystals (JCPDS file no. 27-0699). It is worth noting that, the diffraction peak shifts slightly toward higher angle side as an addition of Mn^2+^. This is mainly attributed to the decrease in unit-cell volume of NaGdF_4_ host because of Mn^2+^ replace of Gd^3+^. Moreover, energy dispersive X-ray spectrometer (EDX) analysis (Figure 1B) shows the presence of Na, Gd and doped Yb, Mn elements, further verifying the substitution by Mn^2+^. In addition, with increasing Mn^2+^ doping content in the product, as shown in Figure 1A. Gd^3+^ content decreases gradually, and the value of Gd/Mn ratio shows a gradual decline compared with the nominal one, whereas Na content keeps unchanged. Because of the incorporation of Mn^2+^ into NaGdF_4_ by substituting Gd^3+^. It should be noted that charge balance in NaGdF_4_ is disturbed after Mn^2+^ replacing Gd^3+^. To maintain charge balance, F^−^ vacancies are formed (Figures 1C,D), which subsequently induce lattice contraction. On the other hand, the ionic radius difference between Gd^3+^ (1.05 Å) and Mn^2+^ (0.96 Å) also results in lattice contraction (Shannon, 1976). Due to these two positive effects, a little shifting to large degree of the diffraction peaks is observed after Mn^2+^ doping.

### Effect on Morphology and Crystal Size of the Products

**Figure 2:**
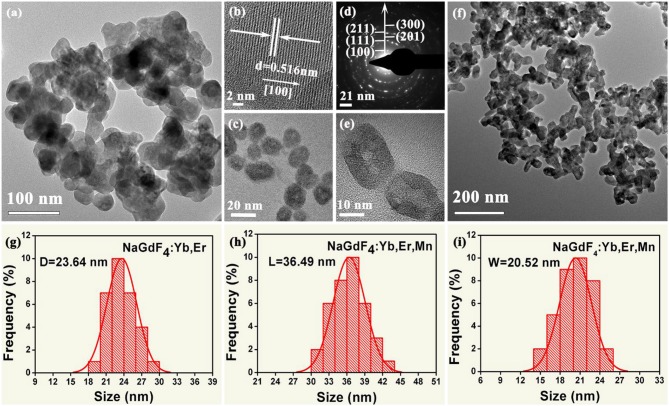
**(a, c)** Low-resolution and High-magnification TEM images of the as-synthesized NaGdF_4_:Yb/Er nanocrystals; **(b)** The corresponding High-resolution TEM (HRTEM) image of a single nanocrystal; **(d)** The selected-area electron diffraction (SAED) patterns of the TEM image shown in **(f)**; **(e)** High-magnification TEM image of the as-synthesized NaGdF_4_: Yb/Er/5 Mn nanocrystals; **(f)** Low-resolution TEM image of the as-synthesized NaGdF_4_: Yb/Er/5 Mn nanocrystals; **(g)** Histograms of particle size distributions for the NaGdF_4_: Yb/Er NCs; **(h)** and **(i)** show histograms of length and width distributions of the NaGdF_4_: Yb/Er/5 Mn NCs, respectively.

To reveal the morphology and size control, we performed transmission electron microscopy (TEM) analyses. As demonstrated in Figure 2, the NaGdF_4_: Yb/Er exhibit irregular shapes (Figures 2a,c), while the NaGdF_4_: Yb/Er/5Mn samples are almost uniformly hexagonal-shaped (Figures 2e,f). High resolution TEM (HRTEM) image (Figure 2b) of a single particle taken from Figure 2c shows the measured interplanar spacing of 5.16 Å, matching well with the (100) crystal plane of β-phase. In the presence of Mn^2+^, the NaGdF_4_ products become hexagonal prisms besides the sphere-shaped NPs, while keeping their hexagonal lattice structure as evidenced by selected-area electron diffraction (SAED) analysis (Figure 2d) taken from Figure 2f. Besides, the average size of products is changed from 23.64 to 36.49 nm/20.52 nm (L/D) (Figures 2g–i) by adding Mn^2+^ with 5 mol% content. A fundamental understanding of the crystal growth process in this work could be mainly ascribed to the substitution of large sized Gd^3+^ (*r* = 1.05 Å) by relative smaller sized Mn^2+^ (*r* = 0.96 Å) (Shannon, 1976). The substitution of Gd^3+^ by Mn^2+^ could generate positive vacancies on the grain surface for the charge balance, subsequently forming transient electric dipoles with the positive poles pointing outward (Chen et al., 2010), which can greatly accelerate the diffusion of F ions from the solution to the grain, therefore promote the growth of NaGdF_4_ UCNPs with increasing Mn^2+^ content.

### Effects of Mn^2+^ on UC Emission Properties of Ln^3+^, Mn^2+^ Doped NaGdF_4_ Nanocrystals

**Figure 3:**
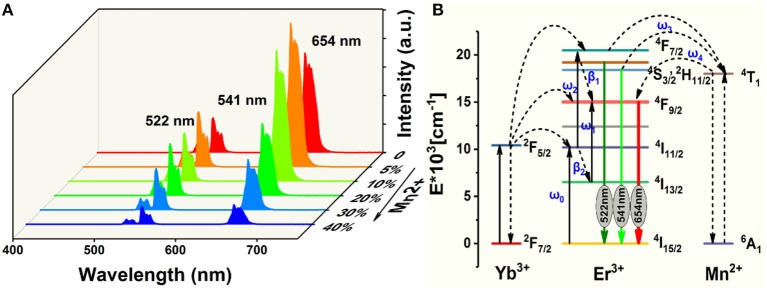
**(A)** Room-temperature UC emission spectra of NaGdF_4_: Yb/Er/*x* Mn (0 mol% ≤ *x* ≤ 40 mol%) nanocrystals under an excitation irradiance of 980 nm laser; **(B)** Simplified energy level diagrams of Er^3+^, Yb^3+^, and Mn^2+^ ions and proposed energy transfer mechanism in NaGdF_4_.

Figure 3A displays the room-temperature UC emission spectra of the irradiated NaGdF_4_: Yb/Er/*x* Mn (*x* = 0, 5, 10, 20, 30, and 40 mol%) nanocrystals under 980 nm excitation, the pump power density is as low as 1.6 W/cm^2^, demonstrating an efficiency of the UC process. All the nanoparticles exhibit three distinct bands in the range of 500–700 nm. Two green emissions ranging from 515 to 535 nm and from 535 to 557 nm were attributed to the ^2^H_11/2_→^4^I_15/2_ and ^4^S_3/2_→^4^I_15/2_ transitions of Er^3+^, respectively. A narrow-band visible emission centered at 654 nm was due to the ^4^F_9/2_→^4^I_15/2_ transition of Er^3+^. Since a close proximity and effective mixing of wave functions of the Er^3+^ and Mn^2+^ ions, there is a high possibility of energy transfer between Mn^2+^ and Er^3+^ ions. And Mn^2+^ itself can't absorb the 980 nm photon, other experimental conditions didn't change. It can be ascribed to non-radiative energy transfer from the ^4^F_7/2_ and (^2^H_11/2_, ^4^S_3/2_) levels of Er^3+^ to the ^4^T_1_ level of Mn^2+^, followed by back-energy transfer (BET) to the ^4^F_9/2_ level of Er^3+^ (Figure 3B) (Sell et al., 1967; Flaherty and Di Bartolo, 1973; Wang et al., 2011; Dan et al., 2016). The BET and cross relaxation (CR) process reduce the population at the ^4^F_7/2_ state which supplies carriers to realize radiative recombination between (^2^H_11/2_, ^4^S_3/2_) and ^4^I_15/2_ states (i.e., green emission). Evidently, the UC emission intensity initially increases with increasing Mn^2+^ doping content under 10 mol%. And then starts to decrease when the Mn^2+^ content is further increased to 40 mol%, which is due to the quenching effect of concentration, and the excess Mn ions increases the distance between Yb and Er ions, thus greatly reducing the effective energy transfer efficiency between Yb and Er. In addition, the up-conversion emission spectra and absorption spectra of NaGdF_4_: Mn, NaGdF_4_: Yb, Er and NaGdF_4_: Mn, Yb, Er in the supplementary document can also assist in proving the energy transfer mechanism between Er^3+^ and Mn^2+^ (Figures S1, S2). The increased ratio of red to green emissions of Er^3+^ suggests a relatively efficient energy transfer process between the Er^3+^ and Mn^2+^ ions, which can be largely attributed to the close proximity and effective mixing of wave functions of the Er^3+^ and Mn^2+^ ions in the crystal host lattices.

In light of all these observations, the following mechanism can be proposed for the upconversion emission in these materials (Figure 3B). Yb^3+^ absorbs the excitation energy and transfers it to Er^3+^ ions, which can be represented as Er^3+^(^4^I_15/2_), Yb^3+^(^2^F_5/2_) → Er^3+^(^4^I_11/2_), Yb^3+^(^2^F_7/2_), with the excess energy being transferred to the surrounding matrix. Er^3+^(^4^I_11/2_) can undergo phonon relaxation to Er^3+^(^4^I_13/2_). Er^3+^(^4^I_11/2_) and Er^3+^(^4^I_13/2_) could populate Er^3+^(^4^F_7/2_) and Er^3+^(^4^F_9/2_), respectively, either by excited-state absorption or by energy transfer from another Yb^3+^ ion. What is noteworthy is that Mn^2+^(^4^T_1_) and Er^3+^(^4^F_9/2_) could be populated through two energy transfer processes, that is (Sell et al., 1967; Flaherty and Di Bartolo, 1973; Wang et al., 2011),

$$\begin{array}{l} \;{\text{Er}}^{3+}{(}^{4}{\text{I}}_{15/2}),{\text{Yb}}^{3+}{(}^{2}{\text{F}}_{5/2})\to {\text{Er}}^{3+}{(}^{4}{\text{I}}_{11/2}),{\text{Yb}}^{3+}{(}^{2}{\text{F}}_{7/2})\;{\text{w}}_{0}\\ \;{\text{Er}}^{3+}{(}^{4}{\text{I}}_{13/2}),{\text{Yb}}^{3+}{(}^{2}{\text{F}}_{5/2})\to {\text{Er}}^{3+}{(}^{4}{\text{F}}_{7/2}),{\text{Yb}}^{3+}{(}^{2}{\text{F}}_{7/2})\;{\text{w}}_{1}\\ \;{\text{Er}}^{3+}{(}^{4}{\text{I}}_{11/2}),{\text{Yb}}^{3+}{(}^{2}{\text{F}}_{5/2})\to {\text{Er}}^{3+}{(}^{4}{\text{F}}_{9/2}),{\text{Yb}}^{3+}{(}^{2}{\text{F}}_{7/2})\;{\text{w}}_{2}\\ {\text{Er}}^{3+}{(}^{2}{\text{H}}_{11/2}),{\text{Mn}}^{2+}{(}^{6}{\text{A}}_{1})\to {\text{Er}}^{3+}{(}^{4}{\text{I}}_{15/2}),{\text{Mn}}^{2+}{(}^{4}{\text{T}}_{1})\;{\text{w}}_{3}\\ \;{\text{Er}}^{3+}{(}^{4}{\text{I}}_{15/2}),{\text{Mn}}^{2+}{(}^{4}{\text{T}}_{1})\to {\text{Er}}^{3+}{(}^{4}{\text{F}}_{9/2}),{\text{Mn}}^{2+}{(}^{6}{\text{A}}_{1})\;{\text{w}}_{4} \end{array}$$

The radiative transfer of the Er^3+^(^2^H_11/2_) and (^4^S_3/2_) to the ground-state (^4^I_15/2_) level gives 522 and 541 nm emissions, respectively, while that of Er^3+^(^4^F_9/2_) to the ground state yields red emission (Figure 3A).

Non-radiative deactivation of Er^3+^ could happen in two ways (Figure 3B). Which could be contribute to the red to green upconversion emission ratio in different samples.

Pathway 1 Phonon relaxation of Er^3+^(^4^S_3/2_,^2^H_11/2_) to Er^3+^(^4^F_9/2_) β1;

Pathway 2 Phonon relaxation of Er^3+^(^4^I_11/2_) to Er^3+^(^4^I_13/2_) β2.

The concentration of Mn^2+^ increases in the appropriate range, the value of w_3_ and w_4_ will increase too. Therefore, R/G will increase. This result indicates that the back energy transfer process of ^4^T_1_ (Mn^2+^) → ^4^F_9/2_ (Er^3+^) is efficient.

To demonstrate the existence of BET and the upconversion mechanism, the excitation power-dependent UC emissions were measured. And the power densities have already been calculated to normalize the UC results. For the unsaturated upconversion process, the number of photons which are required to populate the upper emitting level can be described by the following relationship (Li et al., 2012; Ramasamy et al., 2013):

$$\begin{array}{l} I_{UP}\;\propto \;{I_{NIR}}^{n}\; \end{array}$$

**Figure 4:**
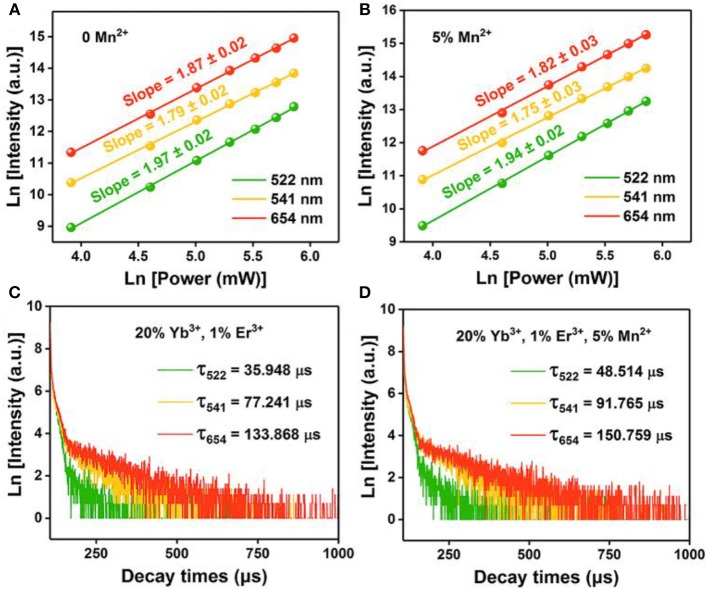
Pump power dependence of the UC emissions in **(A)** NaGdF_4_: Yb/Er UCNPs; **(B)** NaGdF_4_: Yb/Er/5 Mn UCNPs; **(C)**, **(D)** Corresponding decay curves of ^2^H_11/2_ → ^4^I_15/2_ transition (@522 nm), ^4^S_3/2_ → ^4^I_15/2_ transition (@541 nm), and ^4^F_9/2_ → ^4^I_15/2_ transition (@654 nm) of the UCNPs, respectively.

Where I_UP_ is the upconversion luminescence intensity, I_NIR_ is the pump laser intensity, and n is the number of pump photons required. As shown in Figures 4A,B, the slopes of the linear fit of ln(I_UP_) vs. ln(I_NIR_) for the 522, 541, and 654 nm emissions in the NaGdF_4_: Yb/ Er co-doped with 0, 5 mol% of Mn^2+^ ions are all below 2, indicating that two photon processes are involved to produce the green and red UC emissions both in nanocrystals with and without Mn^2+^ ions. Notably, for 5 mol% Mn^2+^ doped NaGdF_4_: Yb/Er nanoparticles (Figure 4B), the slope (n) values for the 522, 541, and 654 nm emissions were 1.94 ± 0.02, 1.75 ± 0.03, and 1.82 ± 0.03, respectively. These values are slightly lower than the values for NaGdF_4_: Yb/Er NPs (Figure 4A). It was reported that a realistic upconversion system that produces detectable upconversion luminescence will exhibit an intensity-vs.-power dependence, which is less than the assumed P^n^. Competition between the upconversion process and linear decay by luminescence to the ground state or relaxation into the next lower-lying state for the depletion of the intermediate excited states results in a significantly reduced slope (Pollnau et al., 2000). A larger upconversion rate means a smaller slope. The result indicates that introducing Mn^2+^ ions can increase the upconversion transition rate leading to the enhancement of upconversion luminescence.

To provide further evidence on the role that Mn^2+^ plays in the enhanced UC emission, the decay curves of the UCNPs with and without Mn^2+^ doping are also drawn in Figures 4C,D, which deviate from single exponential and thus are fitted with the equation proposed by Nakazawa et al. (1999):

$$\tau_{m}=\;\frac{\int_{0}^{\infty }tI(t)dt}{\int_{0}^{\infty }I(t)dt}\;$$

Where τ_m_ is the effective decay time constant, and I(t) is the intensity at time t. It is noted that the decay times of the ^2^H_11/2_→^4^I_15/2_ (@522 nm) transition of the UCNPs have been increased by ~35% after Mn^2+^ doping. Increases of decay lifetime for ^4^S_3/2_→^4^I_15/2_ (@541 nm) and ^4^F_9/2_→^4^I_15/2_ (@654 nm) transition by ~19% and ~13% respectively are also observed after Mn^2+^ doping. For UC materials, a long lifetime usually means a high-efficiency UC luminescence, i.e., a low non-radiative deactivation probability of Ln^3+^ activators. That agrees with the result of UC emission spectra. In this case, the enhancement of BET and prolonged carrier lifetimes indicates that the removal of amorphous surface and the improvement of surface crystallinity (Bian et al., 2018).

**Figure 5:**
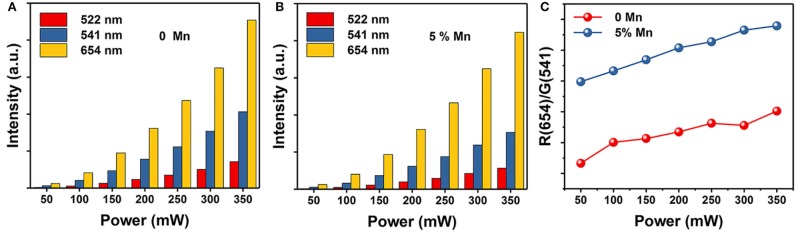
The corresponding integrated UC intensities of green (^2^H_11/2_ → ^4^I_15/2_ and ^4^S_3/2_ → ^4^I_15/2_) and red (^4^F_9/2_ → ^4^I_15/2_) emissions vs. power. **(A)** NaGdF_4_: Yb/Er UCNPs; **(B)** NaGdF_4_: Yb/Er/5 Mn UCNPs; **(C)** Red-to-green ratio of NaGdF_4_ UCNPs as a function of power.

We know that the variation in FIR of two close lying levels of rare earth (RE) ions is due to change in their populations (Wade et al., 2003; Rai, 2007; Brites et al., 2012; Jaque and Vetrone, 2012; Verma and Rai, 2012; Carlos and Palacio, 2016). Due to the energy difference between the ^4^S_3/2_ and ^4^F_9/2_ states is ~2,700 cm^−1^ and follows Boltzmann's distribution (Dong et al., 2007; Dey et al., 2014; Nigoghossian et al., 2017a). The observed FIR variation corresponding to the ^4^S_3/2_ → ^4^I_15/2_ and ^4^F_9/2_ → ^4^I_15/2_ transitions of Er^3+^–Yb^3+^ and Mn^2+^–Er^3+^–Yb^3+^ codoped NaGdF_4_ samples due to change in laser power density generates an idea of optical heating (Debasu et al., 2013, 2016). For experimental verification of the concept of optical heating induced by laser power density, the FIR technique for the same UC emission bands have been used and obtained the factors which affect the change in the intensity ratio. As shown in Figure 5, the UC emission intensity of the two samples are significantly improved with the increase of power density. Moreover, the power-dependent red-to-green ratio increases with Mn^2+^ doping. We can deduce that the red-to-green ratio increases with the increasing of BET process between Er^3+^ and Mn^2+^ (^4^S_3/2_ (Er^3+^) → ^4^T_1_ (Mn^2+^) → ^4^F_9/2_ (Er^3+^)). That is owe to the increasing power density generate an optical heating along with the electrons are largely accumulating at ^4^F_9/2_ state. This result also supports the standpoint that BET process takes part in the up-conversion emissions.

### Temperature Sensor Characterization

**Figure 6:**
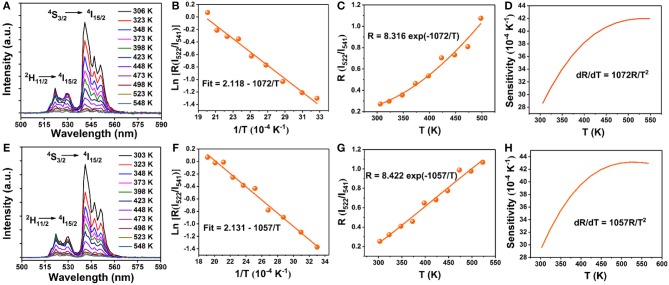
Temperature dependent (303–548 K) normalized UC emission spectra of the **(A)** 0 mol% Mn; **(E)** 5 mol% Mn doped NaGdF_4_: Yb/Er samples in the wavelength range of 500–590 nm; **(B,F)** Monolog plots of FIR as a function of inverse absolute temperature; **(C,G)** The FIR of NaGdF_4_: Yb/Er and NaGdF_4_: Yb/Er/5 Mn phosphors as a function of temperature based on ^2^H_11/2_/^4^S_3/2_ levels; **(D,H)** The relative sensitivity (SR) vs. absolute temperature.

Finally, to explore the possible application of the present investigated NaGdF_4_ in optical thermometry, UC emission spectra of 0 and 5 mol% Mn^2+^ co-doped NaGdF_4_: Yb^3+^/Er^3+^ under 980 nm excitation ranging from 500 to 590 nm are recorded at different temperatures from 303 to 548 K, as depicted in Figures 6A,E. It can observe that these spectra exhibit two distinct emission bands around 522 nm and 541 nm assigned to the ^2^H_11/2_→^4^I_15/2_ and ^4^S_3/2_→^4^I_15/2_ transitions of Er^3+^ ion, respectively. The FIR of these two UC emissions show a remarkable dependence on the temperature (Figures 6C,G), owing to the thermal coupling between ^2^H_11/2_ and ^4^S_3/2_ states of Er^3+^. Based on Boltzmann distribution theory, FIR of two thermally coupled states can be expressed as the following equation: (Chen et al., 2015; Pandey et al., 2015; Nigoghossian et al., 2017a)

(1) $$\text{FIR}=\frac{I_{522}}{I_{541}}=Cexp(\frac{-\Delta E}{k_{B}T})$$

(2) $$\ln\left(FIR\right)=\ln C-(\frac{-\Delta E}{k_{B}})/T$$

where I_522_ and I_541_ are the integrated UC intensities corresponding to the ^2^H_11/2_ → ^4^I_15/2_ and ^4^S_3/2_ → ^4^I_15/2_ transitions, respectively, C is the constant, ΔE is the energy gap between ^2^H_11/2_ and ^4^S_3/2_ states, k_B_ is the Boltzmann constant, and T is the absolute temperature. According to the expression of the FIR, the value of Ln(I_522_/I_541_) vs. the inverse absolute temperature (1/T) is plotted in Figures 6B,F. The slope is fitted to be 1,072 and 1,057, respectively. As a consequence, the energy gap ΔE and the pre-exponential constant are evaluated to be about 745 cm^−1^, 734 cm^−1^ and 8.316, 8.422, respectively. These two parameters are vital factors for the sensor sensitivity (S) of temperature detection, as defined by the following equation (Chen et al., 2015; Pandey et al., 2015; Nigoghossian et al., 2017a):

(3) $$S_{A}=\frac{d(FIR)}{dT}=FIR\left(\frac{\Delta E}{k_{B}T^{2}}\right)=C(\frac{\Delta E}{k_{B}T^{2}})\exp(\frac{-\Delta E}{k_{B}T})$$

The calculated curve of sensor sensitivity as a function of absolute temperature is plotted in Figures 6D,H. It can be seen that the sensitivity keeps increasing in our experimental temperature range, and the maximal value of about 0.0043 K^−1^ and 0.0042 K^−1^ is realized at the temperature of 523 K, respectively. The FIR of NaGdF_4_: Yb/ Er doping with Mn^2+^ is similar to that without Mn^2+^ based on thermally coupled levels (TCL), indicating that the energy transfer between Er^3+^ and Mn^2+^ has a small impact on FIR.

### Optical Heater Properties

**Figure 7:**
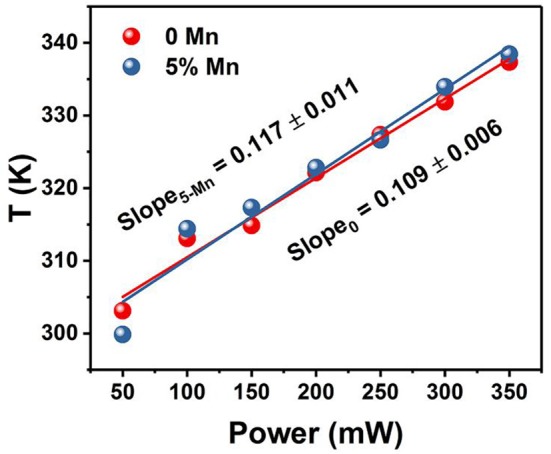
Temperature variation of *x*Mn^2+^, Er^3+^, Yb^3+^ codoped NaGdF_4_ (*x* = 0, 5 mol%) powder as a function of pump power density.

The temperatures at different power densities are calculated through Equation (2). As shown in Figure 7, the temperature exhibits a linearly increasing tendency with the power density increase (Rohani et al., 2015; Nigoghossian et al., 2017b). The slopes of without and with Mn^2+^ doped powders are 0.109 and 0.117, respectively. It could be concluded that optical heat generated by laser power density is mildly more in the Mn^2+^ participate in Er^3+^–Yb^3+^ codoped NaGdF_4_ phosphor than the Er^3+^–Yb^3+^ codoped NaGdF_4_ phosphor. Due to the energy absorbed by the sample may slightly increase as Mn^2+^ doping. As we know, the heat generation inside the samples is due to the non-radiative relaxation involved and the crystalline nature of the synthesized materials. When the energy could not be entirely utilized in the radiative transitions, the excess energy will lead to phonon-assisted non-radiative transitions process, resulting in elevated temperature (Xiang et al., 2014; Hao et al., 2017). The outstanding capability of quickly photo-thermal conversion makes the Mn^2+^ participate in Er^3+^–Yb^3+^ codoped NaGdF_4_ NPs not only a potential candidate as optical heater, but also useful in local hyperthermia based cancer treatment as the temperature were produced within the required range for hyperthermia based treatment (Van der Zee, 2002; Xiang et al., 2014; Lyu et al., 2018).

## Conclusions

In conclusion, a method of transition metal Mn^2+^ doping for the simultaneous morphology/size control, and multi-color output in NaGdF_4_: Yb/Er UCNPs with fixed composition of both host and dopants of lanthanides is demonstrated. The Mn^2+^ dopant makes an enhanced UCL intensity, and larger power-dependent R/G ratio compared to Mn^2+^ free UCNPs. It could be ascribed to the increasing of BET process between Er^3+^ and Mn^2+^ (^4^S_3/2_ (Er^3+^) → ^4^T_1_ (Mn^2+^) → ^4^F_9/2_ (Er^3+^)). Furthermore, the samples show high sensing sensitivities and the maximal sensing sensitivities reach 0.0043 K^−1^ at 523 K. In addition, the input excitation power density induced FIR change and the optical heating of the phosphors have been studied eventually by varying the input excitation power density of the 980 nm NIR diode laser. More importantly, the near-infrared laser induced elevated temperatures (ΔT) reach ~50 K (5 mol% Mn^2+^) when the power density was changed, which is attributed to the increasing phonon-assisted non-radiative transitions process. The internal heat produced from the samples is within the range required for hyperthermia treatment, too. These results provide guidance for the application of Mn^2+^ participate in Yb^3+^–Er^3+^ codoped NaGdF_4_ UCNPs in color modulation, temperature sensing and optical heating.

## Author Contributions

All experimental work was performed by QQ under guidance of YW. All authors contributed to the analysis of the results and to the writing of the paper.

### Conflict of Interest Statement

The authors declare that the research was conducted in the absence of any commercial or financial relationships that could be construed as a potential conflict of interest.
