# Supplementary material for: Effect of Mn2+ on Upconversion Emission, Thermal Sensing and Optical Heater Behavior of Yb3+ - Er3+ Codoped NaGdF4 Nanophosphors
Source: Front Chem. 2019 Jun 6;7:425. doi: 10.3389/fchem.2019.00425 (PMC6562558; doi:10.3389/fchem.2019.00425)
Supplement: Supplementary file 1 [file Table_1.DOCX]

**Supporting Information**

Effect of Mn^2+^ on upconversion emission, thermal sensing and optical heater behavior of Yb^3+^ - Er^3+^ codoped NaGdF_4_ nanophosphors

**Qinping Qiang, Yuhua Wang.***

Department of Materials Science, School of Physical Science and Technology, Lanzhou University, Lanzhou, 730000, China

Key Laboratory for Special Function Materials and Structural Design of the Ministry of Education, Lanzhou University, Lanzhou, 730000, China

*** Correspondence:**

*Corresponding author: Yuhua Wang

Email address: wyh@lzu.edu.cn;





**Figure S1** Room-temperature UC emission spectra of NaGdF_4_: 5mol% Mn, NaGdF_4_: 20mol% Yb / 1mol% Er and NaGdF_4_: 20mol% Yb / 1mol% Er/ 5mol% Mn nanocrystals under an excitation irradiance of 980nm laser.



**Figure S2** The absorption spectra of NaGdF_4_: 5mol% Mn, NaGdF_4_: 20mol% Yb / 1mol% Er and NaGdF_4_: 20mol% Yb / 1mol% Er/ 5mol% Mn samples.

First of all, upconversion emission spectrum shows that almost no upconversion luminescence can be observed in samples doped only with Mn ions. However, Mn^2+^ doping did cause the enhancement of up-conversion emission, in which the enhancement increment of red light was slightly higher than that of green light, that is, the R/G value increased slightly. Thus, increased the population at the ^4^F_9/2_ state. That's what the energy transfer process does, which can be largely attributed to the close proximity and effective mixing of wave functions of the Er^3+^ and Mn^2+^ ions in the crystal host lattices. A similar explanation can be found in literature “Wang, J., Wang, F., Wang, C., Liu, Z., and Liu, X. (2011). Single‐band upconversion emission in lanthanide‐doped KMnF_3_ nanocrystals. *Angewandte Chemie International Edition*, 50(44), 10369-10372”. It fully proves that there is such a process of energy transfer between Er^3+^ and Mn^2+^ as we mentioned. Which can be ascribed to nonradiative energy transfer from the ^2^H_9/2_ and ^4^S_3/2_ levels of Er^3+^ to the ^4^T_1_ level of Mn^2+^, followed by back-energy transfer to the ^4^F_9/2_ level of Er^3+^. In addition, according to literature “Dan, H.K.; Zhou, D.; Wang, R.; Jiao Q.; Yang, Z.; Song, Z.; Yu, X.; Qiu, J. Effect of Mn^2+^ ions on the enhancement red upconversion emission and energy transfer of Mn^2+^/Tm^3+^/Yb^3+^ tri-doped transparente glass-ceramics, Materials Research Bulletin 73 (2016) 357-361.”, it is known that after doping Yb and Mn, Mn has a weak broad emission peak range from 500 to 700nm, whose spectrum overlaps with that of Er. Therefore, the luminescence enhancement after doping Mn in our experiment indicates that there is an energy transfer process between Mn and Er.

According to the absorption spectrum, when only Mn is doped, there is only the characteristic peak of Mn at 276nm. For comparison, after doping with Mn in NaGdF_4_: Yb/ Er samples, the characteristic absorption peaks of Yb and Er at 980nm, 654nm and 520nm are suitably enhanced, respectively. which indicates that there is indeed an energy transfer process between Er and Mn.
